# Mobile health clinics in the United States

**DOI:** 10.1186/s12939-020-1135-7

**Published:** 2020-03-20

**Authors:** Nelson C. Malone, Mollie M. Williams, Mary C. Smith Fawzi, Jennifer Bennet, Caterina Hill, Jeffrey N. Katz, Nancy E. Oriol

**Affiliations:** 1 Harvard Medical School, Boston, USA; 2 Department of Global Health and Social Medicine, Harvard Medical School, Boston, USA; 3 The Family Van, Boston, USA; 4 Department of Anesthesia and Critical Care, Beth Israel Deaconess Medical Center, Boston, USA

**Keywords:** Mobile clinics, Mobile health unit, Health disparities, Social determinants of health, Population health, Health care access

## Abstract

**Background:**

Mobile health clinics serve an important role in the health care system, providing care to some of the most vulnerable populations. Mobile Health Map is the only comprehensive database of mobile clinics in the United States. Members of this collaborative research network and learning community supply information about their location, services, target populations, and costs. They also have access to tools to measure, improve, and communicate their impact.

**Methods:**

We analyzed data from 811 clinics that participated in Mobile Health Map between 2007 and 2017 to describe the demographics of the clients these clinics serve, the services they provide, and mobile clinics’ affiliated institutions and funding sources.

**Results:**

Mobile clinics provide a median number of 3491 visits annually. More than half of their clients are women (55%) and racial/ethnic minorities (59%). Of the 146 clinics that reported insurance data, 41% of clients were uninsured while 44% had some form of public insurance. The most common service models were primary care (41%) and prevention (47%). With regards to organizational affiliations, they vary from independent (33%) to university affiliated (24%), while some (29%) are part of a hospital or health care system. Most mobile clinics receive some financial support from philanthropy (52%), while slightly less than half (45%) receive federal funds.

**Conclusion:**

Mobile health care delivery is an innovative model of health services delivery that provides a wide variety of services to vulnerable populations. The clinics vary in service mix, patient demographics, and relationships with the fixed health system. Although access to care has increased in recent years through the Affordable Care Act, barriers continue to persist, particularly among populations living in resource-limited areas. Mobile clinics can improve access by serving as a vital link between the community and clinical facilities. Additional work is needed to advance availability of this important resource.

## Background

The manner in which people receive health care in the United States has changed substantially over the last decade [1, 2]. As the health care system continues to evolve, it is important to understand the role of mobile health providers. The estimated 2000 mobile clinics that are an integral part of the health care system help ensure access to care for millions and advance health equity [3]. A mobile clinic is a customized motor vehicle that travels to communities to provide health care. They deliver a wide variety of health services and may be staffed by a combination of physicians, nurses, community health workers, and other health professionals. While health care reform has expanded insurance coverage, many barriers to regular health care remain, especially for vulnerable populations [4–6]. Mobile health units help underserved communities overcome common barriers to accessing health care including time, geography, and trust, and have demonstrated improvements in health outcomes and reductions in costs [7–12].

Mobile Health Map, a program of Harvard Medical School, is the only comprehensive database of mobile clinics in the United States [13]. As members of this collaborative research network and learning community, mobile clinics not only supply information about their location, services, target populations, and costs, they also have access to free tools to measure, improve, and communicate their impact.

Using the data supplied by its members, this study describes the mobile health sector including the demographics of clients, services provided, clinics’ geographic distribution, and clinics’ affiliations and funding sources. Preliminary results reported in 2014 for data from 2007 to 2013 included data from 644 clinics [14]. This updated analysis includes data from 2007 to 2017, reflecting updated information from the preliminary report and new additions to the network since 2014, bringing the total to 811 clinics in the network.

## Methods

The aim of this study is to describe the mobile health sector in the United States, including the patients, services, organizational structures, and funding sources. Mobile Health Map was created in 2007 as a partnership between the Mobile Healthcare Association (formerly Mobile Health Clinics Association), Harvard T.H. Chan School of Public Health, and The Family Van, a mobile clinic affiliated with Harvard Medical School. Initial funding was provided by Ronald McDonald House Charities, Harvard University Interfaculty Provost Grant, Boeing Company, the U.S. Department of Health and Human Services, and the Office of Minority Health. The U.S. Human Resources and Services Administration and the Institute for Healthcare Improvement supported the dissemination of the collaboration’s work.

Clinics are invited to join the research network through a variety of recruitment techniques including presentations and exhibits at conferences; emails to the Mobile Healthcare Association members listserv; webinars and conferences sponsored by Institute for Healthcare Improvement, including the 100 Million Healthier Lives program and a U.S. Human Resources and Services Administration webinar series for grantees and grantors; and direct solicitation through online searches. Mobile Health Map consistently ranks among the top results for web searches about mobile health care resulting in many new additions to the network.

**Fig. 1:**
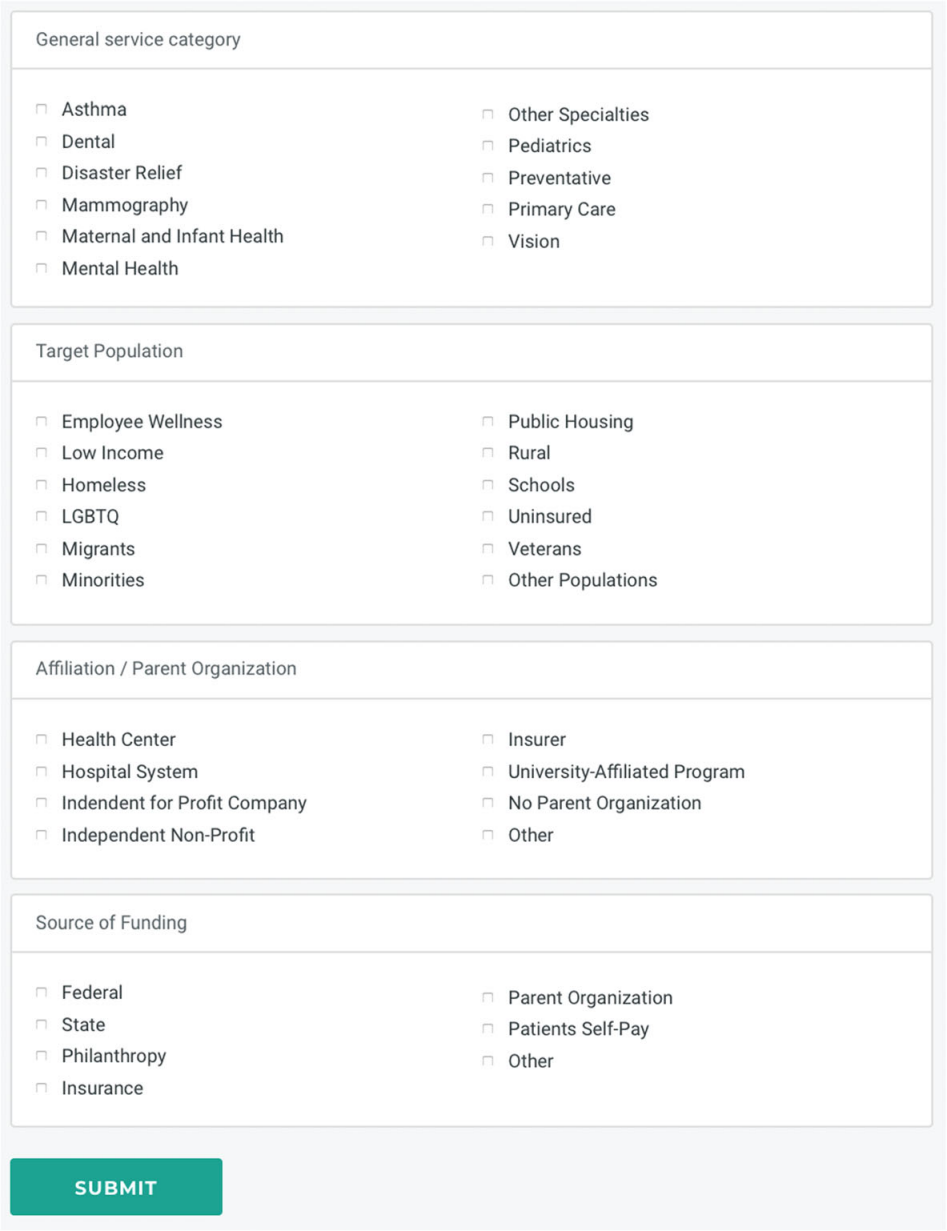
Screenshot of portion of New Clinic Entry form

Of the estimated 2000 clinics around the country, a total of 811 had joined the network as of April 24, 2017, including 167 new clinics since 2014. Mobile clinics voluntarily submit data to Mobile Health Map’s website using a series of online forms [13] and provide information about their organization, operations, services, and clients (Fig. 1). Clinics are asked to report on all indicators to the best of their knowledge as frequently as possible on an annual basis. To ensure the accuracy and consistency of the information provided, the Executive Director of Mobile Health Map and volunteers under her supervision had phone conversations with approximately 10% of clinics.

The database includes information on clinic office locations (city, state, country, and zip code). Clinics designate their target populations (employees, LGBTQ, public housing, schools, minorities, migrants, veterans, rural, homeless, low income, uninsured, and other populations). Clinics indicate the percentage of clients falling in various categories of age (0–17, 18–44, 45–64, and 65+), gender, and insurance status (Medicaid/Children’s Health Insurance Program, Medicare, private, and uninsured). They provide data on the types of care offered (prevention, dental, primary, mammography, maternal and infant health, pediatric, asthma, mental health, disaster relief, and other). The cost of operations, affiliations (independent/for profit, no parent organization, faith-based, insurer, health center, independent/non-profit, university-affiliated, hospital, and other), and funding sources (from parent organizations, states, patients self-pay, public insurance, private insurance, federal, philanthropy, and other sources) of clinics are also provided.

The data presented represents the data collected over the entire 10-year period (2007–2017), with only the most recently updated data presented for each clinic if they reported during multiple years. All data are aggregated across the mobile clinic’s population and do not include individual patient information. Results were compared to a preliminary analysis from 2014. If a clinic reported updated information since 2014, the updated information was used in our analysis. However, if a clinic has not reported since that time period, the insurance status of clients, for example, when they did report, was still used in our analysis.

### Analysis

Clinic services, cost, and client demographic data were exported from Mobile Health Map website into Microsoft Excel. Aggregate client demographic information from the online impact and quality tools were uploaded to Qualtrics [15] and subsequently exported into Microsoft Excel. The estimated proportions across all clinics were derived from an unweighted mean of the estimate measurements provided by each individual clinic. For example, if clinic A reported that they served an estimated 50% Asian clients and clinic B estimated an average of 30% Asian clients, the reported average per clinic would be 40% Asian clients. This approach was used because the goal of the analysis was to provide averages across clinics with the clinic being the unit of observation, rather than summarizing data at the individual level. While the use of a weighted average was considered to give greater weight to clinics with higher volume, this type of analysis was not possible because only 168 of the clinics provided total patient volume. The number of clinics reporting on each variable differed and is depicted in the text, figured, and tables.

## Results

### Affiliations and funding sources

**Fig. 2:**
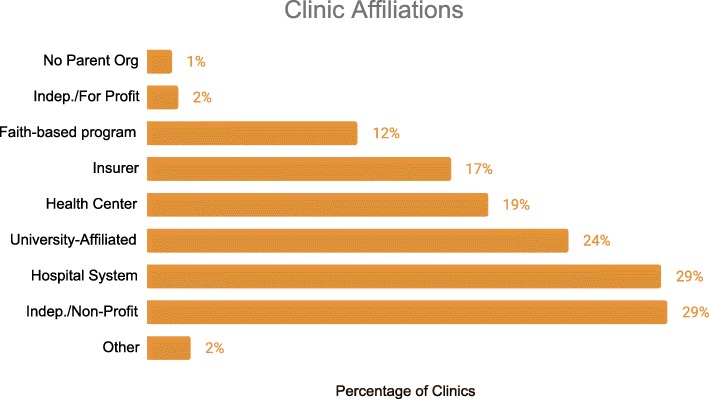
Mobile Health Clinics Affiliations. 286 clinics report on the affiliations or parent organizations. *Clinics may designate multiple affiliations

Though 33% of the 286 reporting clinics were independent programs, mobile clinics are often part of a larger organization. The most common of these affiliations were with hospital systems in 29% and universities in 24%. Nineteen percent (19%) reported to be affiliated with health centers, 17% with insurance companies, and 12% with faith-based organizations (Fig. 2).

Of the 173 mobile clinics reporting on total annual cost, the average cost per mobile clinic operation was $632,369. Of the 58 clinics designating themselves as a prevention clinic (excluding primary care), the average cost was $319,868. Of the 58 primary care clinics reporting cost, the average was $981,092. The average cost of the 37 dental clinics reporting was $1,169,559.

**Fig. 3:**
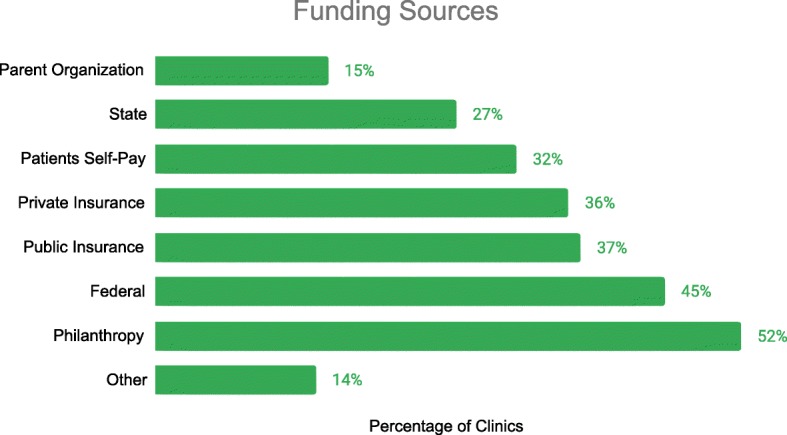
Reported Sources of Funding for Clinics. 281 clinics report on the sources of their funding. *Clinics may choose multiple sources of support

To cover these costs, mobile health clinics depend heavily on philanthropy and government funding. Of the 281 clinics reporting on their sources, 52% reported philanthropic support and 45% reported federal funding (Fig. 3). Health insurance companies also provide much of the funding: 38% reported revenue from public insurance providers and 37% from private insurance providers. Thirty-two percent of clinics reported client payments as a source of revenue.

**Fig. 4:**
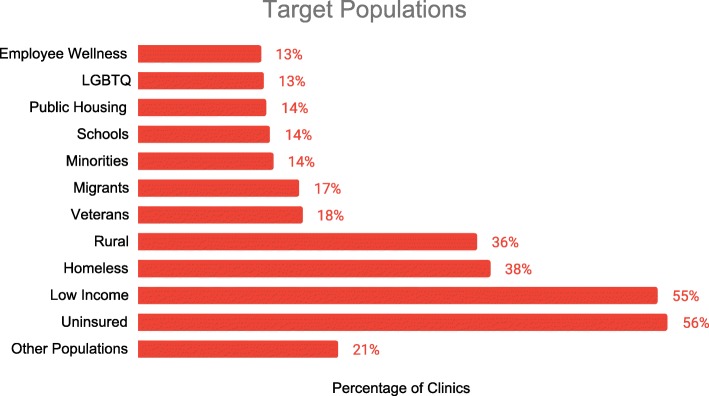
Two hundred ninety-one mobile clinics report on the specific groups they target. *Clinics may select more than one option

### Client demographics

A variable number of clinics reported on each data element. Of the 253 mobile clinics that reported information on the number of visits, the median number of annual visits was 3491 with an interquartile range (IQR) of 1828 to 6050. One hundred sixty-six clinics also reported the number of new visitors annually, of which the median was 1200 new visits with an IQR of 574 to 2123.166 clinics also reported the average number of new visitors annually, of which the average was 2713. One hundred ninty-two clinics reported gender distribution. Female clients make up a slight majority with each mobile clinic serving an average of 55% female clients and 44% male clients. A category for transgender people was recently added.

Of the 183 clinics that reported on age, the average percentage of clients between 0 and 17 years of age was 41%, and the average percentage falling between 18 and 44 years was 20% per clinic (Table 1). The average percentage of clients 45–64 years of age was 31% per clinic, and the lowest utilization is found in the age group 65+ with an average 11% per clinic.

**Table 1:** Age and Race/Ethnicity Distribution of Mobile Clinic Clients. 183 and 186 mobile clinics’ estimates of the percentage of clients visiting their clinics annually stratified by age and race/ethnicity, respectively

**Age**	**Average Percentage of Clients**
0–17 years old	40.9%
18–44 years old	20.0%
45–64 years old	30.9%
65+ years old	10.5%
**Race/Ethnicity**	**Average Percentage of Clients**
White (not Hispanic / Latino)	41.5%
Black / African American	35.3%
Hispanic / Latino	26.6%
Mixed / Other	4.1%
Asian	2.8%
American Indians and Alaska Natives	1.8%
Native Hawaiians and other Pacific Islanders	1.0%

One hundred eighty-six clinics provided data on the race/ethnicity of clients. The average percentage of white clients was 42%, Black/African-American clients was 35%, and Hispanic clinics was 27% (Table 1**)**. The average percentage of Asians, Native American, multiracial, and those clients designating their ethnicity as “other” were less than 5%**.**

Many mobile clinics aim to reach populations with limited access to care. To understand which client populations the clinics were designed to serve, clinics are asked to report the group or groups they target. Of the 291 clinics reporting, 56% targeted the uninsured, 55% low-income groups, 38% homeless persons, and 36% rural communities (Fig. 4).

Of the 146 clinics that provided information on insurance status of clients, the average percentage of uninsured clients was approximately 41%. The average percent of clients covered by Medicaid/CHIP was 30% per clinic and by Medicare was 15% per clinic. The average reported percentage of clients with private insurance was 25% per clinic, some of whom also have coverage with public insurance.

### Services offered

**Fig. 5:**
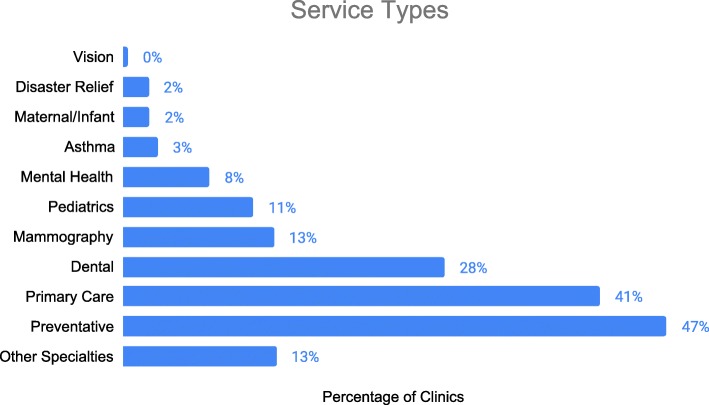
Services Reported by Mobile Clinics. 724 clinics reported on the serve type of their clinic. The “other specialty” category includes vision, asthma, maternal and infant health, disaster, homelessness, and other services

Mobile clinics have the option to report on the type of services they offer. Of the 724 clinics that provided information on service type, 47% reported that they provide prevention services exclusively, 41% reported to be primary care focused, and 28% reported to provide dental care (Fig. 5). Mammography, pediatric, mental health, asthma, maternal and infant health, disaster relief, vision, and other specialty services are also provided by mobile clinics.

Of the 67 clinics reporting on specific services/screenings provided, 31% provided screening for hypertension, 31% provided diabetes screenings and treatment (or referral to treatment), 28% provided diet counseling, and 27% provided cholesterol screenings. The mobile clinics also offer screening for colorectal (13%), cervical (13%) and breast cancer (19%) and osteoporosis (9%). There are also services for hearing (6%), vision (13%), depression (25%), and obesity (25%). Clinics describe discussion of daily aspirin use (13%), calcium supplementation (12%), and folic acid use for women of childbearing age (13%). Smoking cessation advice (25%) and alcohol screening and brief counseling for alcohol use (25%) are also provided on some clinics.

### Geographic distribution

**Fig. 6:**
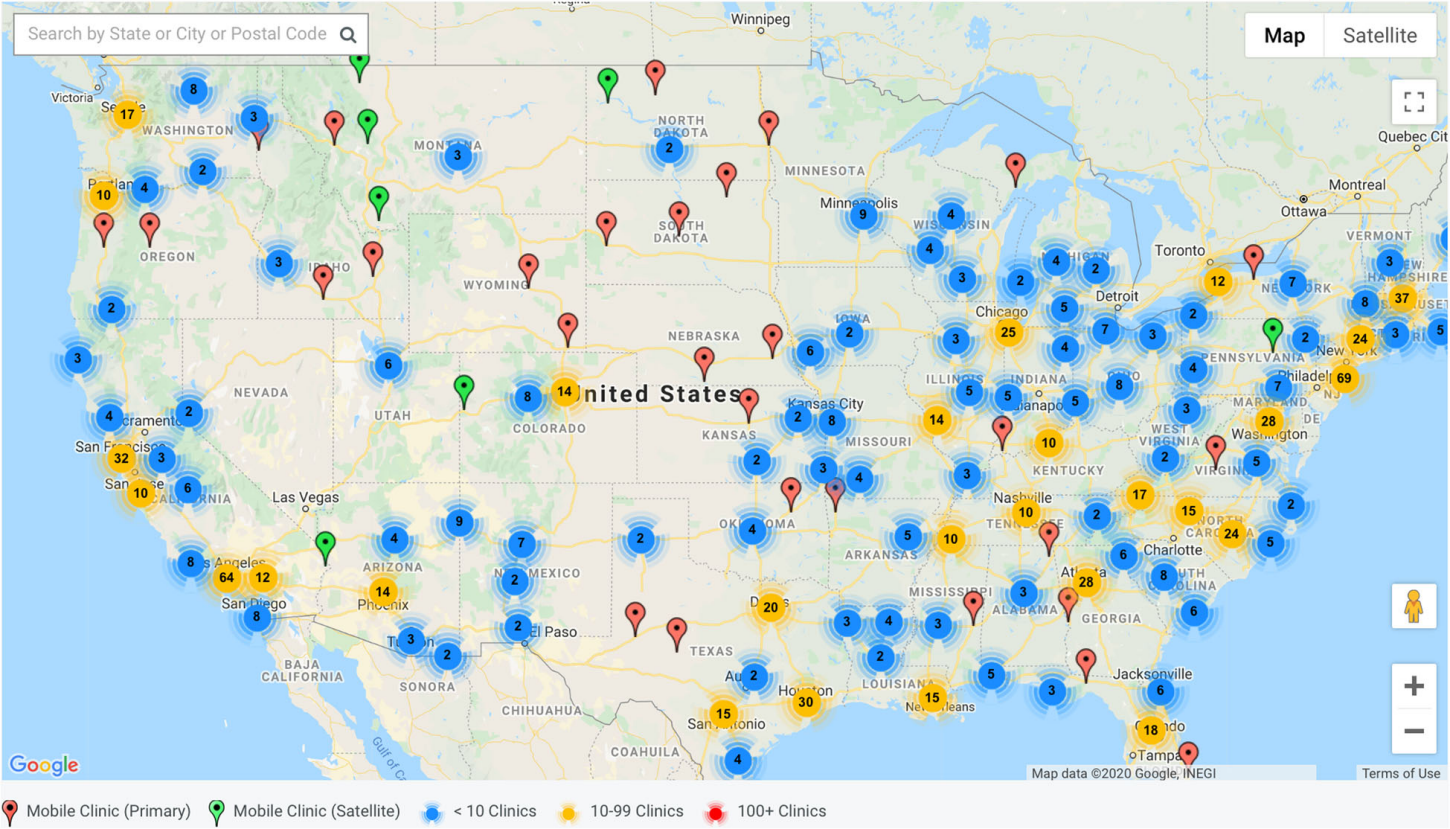
Mobile Health Clinics Operating in the United States of America. This figure is a May 2017 screenshot of mobile clinics mapped by MobileHealthMap.org with 811 clinics representing all 50 states, the District of Columbia, Puerto Rico, and other nations globally. The red markers denote a single clinic. Circles represent a concentration of clinics (blue: < 10, yellow: 10+)

Mobile clinics operate in all 50 states, the District of Columbia, and Puerto Rico (Table 2). Many are located in areas of high population density in major cities around the country such as Atlanta, Boston, Chicago, Dallas, Denver, Houston, Los Angeles, New York, Orlando, Phoenix, San Francisco, San Jose, Seattle, and St. Louis (Fig. 6). There are also clinics located in less populated areas.

**Table 2:** Number of Mobile Health Clinics by State**.** This table shows the number of mobile health clinics represented by each state on the Mobile Health Map

Northeast		Midwest		South		West	
Connecticut	6	Illinois	25	Florida	36	Arizona	21
Maine	7	Indiana	5	Georgia	16	Colorado	12
Massachusetts	29	Michigan	13	Maryland	14	Idaho	4
New Hampshire	4	Ohio	13	North Carolina	41	Montana	4
Rhode Island	3	Wisconsin	8	South Carolina	12	Nevada	1
Vermont	1	Iowa	2	Virginia	15	New Mexico	7
Delaware	3	Kansas	13	District of Columbia	3	Utah	2
New Jersey	9	Minnesota	9	West Virginia	4	Wyoming	2
New York	61	Missouri	15	Alabama	7	Alaska	2
Pennsylvania	16	Nebraska	4	Kentucky	11	California	120
		North Dakota	3	Mississippi	7	Hawaii	13
		South Dakota	3	Tennessee	15	Oregon	13
				Arkansas	5	Washington	20
				Louisiana	23		
				Oklahoma	5		
*Puerto Rico	1			Texas	61

## Discussion

Mobile health clinics deliver care to some of the country’s most vulnerable populations, including people of color, people experiencing poverty and/or homelessness, the uninsured, rural communities, veterans, and immigrants. They are located across the United States and operate with the financial support of health care systems, universities, philanthropy, and government agencies.

The people and organizations that operate mobile clinics are often motivated by a commitment to underserved communities and use the mobile clinic as a vehicle to deliver care in ways that differ from traditional medical settings. For example, Bouchelle and colleagues reported that The Family Van, a mobile clinic in Boston, creates a culture of respect and inclusivity [16]. In many instances, mobile clinics serve as a bridge between communities and the health care system. As health care leaders and policymakers increasingly recognize the importance of social determinants of health and community-clinical linkages, mobile clinics are well-positioned to further these goals.

Using the median number of annual visits (3491) and the estimated 1500 to 2000 mobile clinics nationwide, we estimate 5.2 to 7.0 million visits to mobile health clinics each year. Assuming the lower estimate of 5.2 million visits, there are an estimated 2.1 million visits by uninsured persons, 2.3 million visits by publicly insured persons, and 2.1 million visits by children to mobile health clinics.

Mobile clinics saw a greater percentage of people of color than then the general U.S. population. For example, in the 2010 U.S. Census 13.4% of respondents reported a race of Black or African American. In contrast, the average percentage of Black or African American clients in this sample is 35%. In the same census, 18.3% of residents identified as Latino or Hispanic. In this group of mobile clinics, the average percentage of Latino or Hispanic clients was 26.6% [17].

Comparing the results of this analysis to those of the initial data collection published in 2014, it is notable that the percentage of people attending mobile clinics that were uninsured dropped from 57 to 40%. This drop may be due to the implementation of the Affordable Care Act. It suggests that many mobile clinics that previously served uninsured patients adapted to the new health care environment and are able to bill insurance for visits that were previously supported through other means, like philanthropy.

Mobile health clinics straddle the community and health care system. They often address important social determinants of health including food and housing insecurity, education, and job opportunities. By collaborating with local agencies such as churches, community health centers, and other hospitals and clinics, mobile clinics connect community members with both medical and social services.

Though mobile clinics operate all over the country, they are commonly located in densely populated cities. There is a lack of clinics in the rural parts of every state and many parts of the Midwest, and as a result, populations that may continue to be void of adequate access to health care and areas where mobile clinics can have an impact.

Mobile Health Map is a powerful tool for the mobile health sector to understand itself and demonstrate its role in the greater health care system. This information can help policy makers, payers and providers understand the services they provide and the vulnerable populations they serve. Understanding this will help clinics advocate for their role as a critical part of the health care safety-net and experts in community-clinic linkage.

Mobile Health Map is a pioneer in the mobile health sector and is the source of much of the existing scholarship in the area [3, 14, 18]. The database allows researchers and practitioners to monitor and evaluate the sector and its impact. Further research is needed to understand the changing role of mobile health care in value-based payment models, as well as how mobile health providers integrate behavioral health services.

## Limitations

Of the estimated 2000 mobile clinics around the nation, 811 were registered with the Mobile Health Map as of April 24, 2017. These programs are self-selected and self-reporting. Because there is no similar database of mobile clinics, it is not possible to evaluate the representativeness of our sample. While some clinics outside the U.S. participate in Mobile Health Map, they are not the primary audience and as such, this analysis is also limited to clinics in the U.S. However, for a health care sector previously uncharted, this sample gives us a broad picture of the state of mobile health in the U.S.

Many clinics provide the address of the location where they are headquartered rather than the individual communities they serve. As a result, the breadth of communities served, and thus the true scope of the sector, is likely underestimated by this study. All data provided about costs are self-reported and clinics may differ by the types of costs (e.g. operating, capital) they include.

Given the provision of care for primary and secondary prevention available at mobile clinics, it is highly likely that health outcomes can improve with concomitant cost savings. Therefore, future studies should consider these outcomes. Despite these limitations, the results of the study provide insight on the innovative ways mobile clinics expand the boundaries of the health care system and improve health equity, especially among our most vulnerable populations.

## Conclusion

With an increasing emphasis on population health and meeting people where they work, live, and play, understanding why and how these systems operate can inform effective community-clinical linkages. While mobile clinics exist across the country, many underserved rural areas and under-resourced urban areas continue to suffer from health disparities that could be addressed by expanding the network of mobile clinics.

Mobile clinics reduce barriers to health care including transportation, time, system complexity, and trust. They are an integral part of the health care system and are supported by government agencies, insurance companies, and philanthropy. To advance health equity and reach the most vulnerable and disenfranchised populations, we need to increase investment in mobile clinics and other innovative ideas that promote preventive services and expand the boundaries of the traditional health care system.

## Data Availability

The datasets generated and/or analyzed during the current study are not publicly available due to clinic privacy, but aggregated data about members is available from the corresponding author upon reasonable request.

## Abbreviations

CHIP: Children’s health insurance program
LGBTQ: lesbian, gay, bisexual, transgender, transsexual, queer
